# Professional Ethics

**Published:** 1880-02

**Authors:** J. J. Birge


### PROFESSIONAL ETHICS.

BY J. J. BIRGE.

The science of ethics germinated soon after our progenitors, Adam and Eve, partook of the forbidden fruit in the garden of Eden. The first out-cropping of which we, at this late date, have any knowledge, was in the use of the fig-leaf; for, after the eyes of

Adam and Eve were opened, they saw that they were in a state of nudity; and after due consultation, their newly acquired perception of true refinement and modesty prompted them to make from the fig-leaf, aprons to wear, in order that they might appear in public in more becoming style.

Since that early day there have been many and very great changes in regard to custom, manners, dress, etc., among most of the nations of the earth. But I was a little surprised recently, on meeting a lately returned traveler from the interior of Japan, to learn that at the fashionable watering places in the heart of that country, during the hours of bathing, they stand just where our progenitors did before they ate of "the tree of knowledge;" for the two sexes, literally in the garb of nature, walk down to and into their bathing tubs, some of which are large enough for eight or ten to bathe in at the same time, others smaller. I am told that after the two sexes have gone through their ablutions together, which consume some time, for they chat and play in the water and splash it over each other as our children would, they leave the baths and walk to the verandas of their houses, and there bask in the sun's rays in the most *nonchalant* manner possible, keeping up a cheerful and animated conversation, until the soft balmy air and the sun have nearly vaporized and drank in the pearly drops that were gently trickling down their nude, and in many cases most beautiful forms. They then dry with towels and dress. I am told that this is all done with the innocence of children, and without the least shadow of thought that there is in it the slightest impropriety. With us, at our watering places, such a custom would mantle many cheeks with a crimson blush.

We find, indeed, even among the most civilized nations, very great diversity as regards the prevailing system of social ethics, and the resulting rules of etiquette. But Mr. President, I believe that our Divine Maker, has given to every intelligent human being, at least a crude code of ethics. It is innate, and if we could cherish that ethical instinct so implanted within us, and guard sacredly and conscientiously against any violation of it, it would accomplish more good, relieve more suffering, fill more hearts with joy, and do more to elevate the human race in all that is desirable—morally and physically—than all the precepts, laws or edicts that have gone forth since the dawn of civilization. In fact, we might then look for the long desired millennium. But, unfortunately, this is not the case. We have made, it is true, great advances in civilization. In natural science, our progress has been great. We can measure the heavenly bodies which, in distance, are billions of miles from our earth, and we can almost analyze their composition. By the aid of electricity, San Francisco may to-day exchange greetings with any of the great cities upon our globe; Edison and other scientists are making use of the same subtile force, to dispel the darkness of night, and almost give us day instead; and a thousand other strides in progress; might here be noted, would time permit. Yet, unfortunately, in the science of moral philosophy, or a system of moral principles, truly observed and carried out, we are to-day, sadly deficient. The same evil spirit that led to the fall of man still clings to human nature, impairing the force of the better impulses, and checking moral advance. Our progress in this respect is very slow. Indeed, many think we are retrograding, and that the science of dishonest money-making, seems to absorb the minds of too many who, by education and social standing, claim position in respectable society; but who, were they really to answer for their crimes, would occupy a prisoner's cell with fit companions, thereby becoming powerless for further imposition upon the credulous, or for increasing the list of the widows and orphans who mourn the desolation of their homes.

"Professional Ethics:" What does it demand of us—of the dental profession? Paramount to all other things, is a careful and exact fidelity to our patients. We should be prepared to throw upon them all that is known to science in our profession. To do this, we should not only become well informed in anatomy, physiology and thereapeutics, but we should keep up with the dental literature of the day; we should analyze and test the gems which are, from time to time, added to it through the journal and the latest standard works of this country as well as those of Europe. By so doing we obtain the benefit of the careful researches of the greatest minds in our own profession, as well as in that of medicine; information, which they have consumed many of their best years in obtaining, and in testing by analysis, by the use of the microscope, and by careful watching of therapeutic effects.

We have to combat with the subtle forces that are wasting away the teeth of the young, as well as the various diseases of older persons. Those who submit themselves to our charge, have a right to expect careful and scientific treatment in all that we undertake; and if we fail through a lack of earnest effort in that respect, we are guilty of grossly unprofessional conduct, and richly deserve the contempt of both the profession and the public, and sooner or later it will come.

Our office rooms should be kept scrupulously clean; and particularly our instruments, napkins, and all appliances which may come in contact with the patient; and, if possible, our finger-nails should be kept out of mourning. Even amid the most trying difficulties, the dentist should be kind, gentle, affable and patient. If he should meet with a mishap, as, for example, should break a tooth or a forceps, he should not fly into a rage and dash the instrument across the room, endangering the lives of those present and presenting to them the appearance of one just escaped from the mad-house. I happened, on a certain occasion, years since, to witness an outburst of that kind. If I had not been good at dodging, I should most likely not have been here to-day to bore you with this paper, for my skull would probably have been a sacrifice, and the flying forceps would no doubt have finished my earthly career.

What is our professional duty toward each other? We may sum it up almost entirely in one word—*integrity.* Let that be our guide and we shall seldom do injustice to any member of our profession. We should be slow to believe unprofessional things of our brother members, which may be told us by new patients; especially of a member in good standing. A certain class of patients, when they come to us, will often reflect in some way upon their former dentists, thinking that disparaging words spoken of a rival will be pleasing. Another class, more refined, do not intend to do injustice, but they receive wrong impressions—"get things mixed,"—will say that such and such a dentist said so and so, or did thus and so; often things which we know full well, it would be impossible for any intelligent dentist to say or do. It may be of bad fillings or of extracting a wrong tooth, as, for example, a permanent molar, assuring the patient that it was a deciduous one, and the patient is perhaps really mourning over an imaginary injury which is fancied sure to extend through life. Now when a dentist, in his greed for money, knowing these impressions to be entirely without foundation, helps to strengthen and confirm them, he is the assassin. He does not, it is true, strike a blow in the dark, destroy life and stain his hands with human blood; but he stands concealed with an upraised gleaming blade, ready to strike down and destroy both the professional and the moral standing of his fellow dentists. And for what purpose? That he may put a few more paltry dollars in his own pocket!

A man's honor should be more dear to him than his life; and it is somewhere said that he who would undertake to destroy an honorable reputation is more guilty than he who actually takes life. In one case the guilt has a limit in the death of its victim; in the other, it is almost without limit, for it not only destroys the victim of to-day, but its effect passes on through future generations to blacken and defame his name so long as history and civilization remain. I have always thought that the slanderer and defamer of character should stand thus convicted by all honorable men.

These assassins of character steal upon their victims in various ways. They use a diversity of weapons to accomplish their aim. Sometimes it is by innuendo, sometimes by direct insinuation. Perhaps it is merely a shrug of the shoulders; or again, "they don't wish to say anything." At other times, deep, and even fiendish schemes are concocted, and boldly carried out. Probably few of us have escaped entirely uninjured, from these poisoned shafts which are hurled, with such deadily aim, by some members of our profession.

What a different being is the truly honorable professional man! He stands out in bold relief from the above class, and in strong contrast with it. He is ever ready to extend the helping hand to the young dentist, or to the weak in the profession, who are earnestly seeking information, knowledge and advice. His generous impulses, his keen perception of professional honor, will as quickly stir him with indignation, and bring burning words from his very heart to defend an honorable brother from unjust aspersions, as to defend his own honor were it similarly attacked. If, in the teeth of a new patient, he finds evidence of difficult, skillful and artistic operations previously performed, he is delighted; he examines them with the eye of an artist, and feels that such work is an honor to the profession, as well as to the dentist who so faithfully fulfilled his duty. He will surely make inquiries, and learn the name of the fortunate man who did the work; and he delights to speak in the most eulogistic and complimentary terms of it to his patient. The finest painting by one of "the old masters," can have no greater effect upon the most enthusiastic artist of the present day, than does a masterpiece of art produced by one eminent in *our* profession upon the truly appreciative member of it.

What does professional ethics demand of the young graduates, who have listended to the teachings of the honored professors in our dental colleges, where, before they received their diplomas, they are admonished to guard the dignity of their profession? to be guilty of no act which can reflect upon themselves as honorable men; to descend to no quackery, or to charlatanism in the way of advertising "cheap work," "new modes of practice," and humbuggery in general; and to which admonitions they tacitly assent, and thereby virtually pledge themselves to strictly carry out.

It demands of them, if they have one spark of honor in their composition, strict compliance with the rules laid down; respect for their preceptors, their professors, their alma mater, and the profession; that they should not perjure themselves as soon as they are fairly beyond the shadow of the college which has honored them with a diploma. But, Mr. President and gentlemen, it is most humiliating to honorable men of the profession, to see *graduates* drop into the gutters, and become the professional companion of the class of men found there. I sometimes think it is in the blood, inborn; and that, if an attempt be made to lift such persons from the gutter by education, they will, the moment you let go of them, be almost sure to drop back into their native elements, relinquishing all noble aspirations, and content again to mingle with the common herd.

One word in regard to dental associations: We do not claim that all the members of such associations are as worthy as they should be. But suppose we should divide our profession, here in the United States, into two parts, the first composed of those only who belong to dental societies, and the other of those entirely outside of such societies. Can any one doubt for a moment, the vast difference between them as regards intelligence and respectability, or moral and professional standing? I think not.

To the first, we may say, would belong all of the great and leading spirits of our profession; those who, by their teachings, their clinics and their pens, have done as much during the past thirty years to build it up; all the professors of our dental colleges; the men who, by their deep researches, by the use of the microscope, the delicate tests of chemistry, and by patient and unceasing efforts in all the scientific branches of our profession, have given to the American dentists, in all civilized parts of the world, a pre-eminence rarely possessed by those of any other nation.

Now, let us see whom we find outside of our societies who claim to belong to the profession. There are a few for whom we have a great respect; but, are there not, even among those few, some who, vulgarly speaking, may be called, "old fogies?" who, years since, so far as progress is concerned, came to a stand-still and have run along in the same groove in which they were found twenty years ago? They seem to be clad in an impenetrable armor. The bright ray of light thrown out by science, which penetrates the soul of others, and electrifies them to new exertions, has no effect upon this class. Most of them still cling to their antiquated notions, and, of course, with such there can be no progress.

Now, Mr. President, who are the balance of this last party? They are the class who advertise "cheap dentistry;" who advertise to do work for what would be less than one-half the cost of *the material alone,* if the work were done as it should be done. This class inflict irreparable injury upon the public; more particularly upon that credulous, unthinking, ignorant portion of it, who are duped by such advertisements. In fact, all the charlatans, the quacks, the humbugs and the cheats, who have crawled from the gutters of society into our profession, are there. No, not all, for two of the most noted of that class, who disgraced our profession, and their holy religion as well, while on this coast, are *not* among them. We learn that one of these two is in the State Prison of Massachusetts, and the other in that of North Carolina.

It is unquestionably desirable that every dentist of respectable reputation, should connect himself with some dental association. He is, otherwise, in danger of losing that respectable reputation. The fact is that he does not realize this danger. He does not properly consider the position which he occupies, in allowing himself to be classed with those who have not so associated themselves. It is said that "a man is known by the company he keeps." These gentlemen are not sufficiently alive to the character of the "company" with which they are likely to be identified. I have often thought that if there could be a grand review of the profession, divided, as I have above suggested, into two parts, arranged in two lines, which we may term respectfully "the associates" and the "non-associates," with the entire public as spectators, and the really excellent men of the "non-associates" were to step out of their line, advance a few feet, about face, and take a "square look" at the balance of said line, they would at once awake to "the situation;" would realize the false position which they occupied in permitting themselves to be classed with such a crowd; and, feeling the effect of the public eye, instinctively conscious of the popular verdict then and there silently rendered, would hasten to rectify that false position, and enroll themselves, without delay, in the ranks of the associates.

In conclusion, Mr. President, I will simply ask this question: Would it not, in this view of the subject, which I think is the correct one, be more *professional* for every practitioner of dentistry to attach himself to some dental society? *He* would certainly be the gainer by so doing; and he could undoubtedly benefit others in return. All respectable dentists are cordially invited to join our societies; and it seems to me that "professional ethics" require an acceptance of the invitation so warmly extended to them.

